# The Application of Cell-Free Protein Synthesis in Genetic Code Expansion for Post-translational Modifications

**DOI:** 10.3389/fphar.2019.00248

**Published:** 2019-03-20

**Authors:** Sumana Venkat, Hao Chen, Qinglei Gan, Chenguang Fan

**Affiliations:** ^1^ Cell and Molecular Biology Program, University of Arkansas, Fayetteville, AR, United States; ^2^ Department of Chemistry and Biochemistry, University of Arkansas, Fayetteville, AR, United States

**Keywords:** genetic code expansion, cell-free protein synthesis, non-canonical amino acid, post-translational modification, protein phosphorylation, protein acetylation, protein methylation

## Abstract

The translation system is a sophisticated machinery that synthesizes proteins from 20 canonical amino acids. Recently, the repertoire of such composition has been expanded by the introduction of non-canonical amino acids (ncAAs) with the genetic code expansion strategy, which provides proteins with designed properties and structures for protein studies and engineering. Although the genetic code expansion strategy has been mostly implemented by using living cells as the host, a number of limits such as poor cellular uptake or solubility of specific ncAA substrates and the toxicity of target proteins have hindered the production of certain ncAA-modified proteins. To overcome those challenges, cell-free protein synthesis (CFPS) has been applied as it allows the precise control of reaction components. Several approaches have been recently developed to increase the purity and efficiency of ncAA incorporation in CFPS. Here, we summarized recent development of CFPS with an emphasis on its applications in generating site-specific protein post-translational modifications by the genetic code expansion strategy.

## Introduction

**Figure 1:**
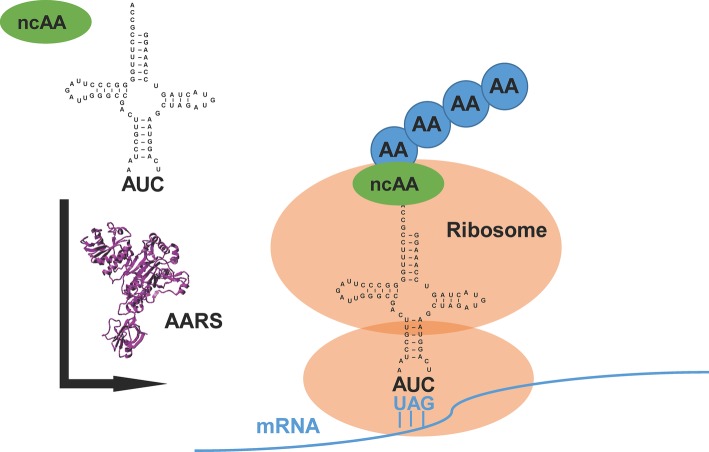
An overview of genetic code expansion. The evolved aminoacyl-tRNA synthetase (AARS) charges the tRNA with the non-canonical amino acid (ncAA). The tRNA harbors the CUA anticodon, which can read the introduced amber stop codon UAG in the mRNA, bringing the ncAA at the designed position in target proteins. AA: canonical amino acid.

The translational machinery in nature provides a new avenue of chemistry in which synthetic biologists could prospect beyond the repertoire of 20 canonical amino acids (Liu and Schultz, 2010). Generally, an orthogonal pair of aminoacyl-tRNA synthetase (AARS) and its cognate tRNA, which does not cross-react with endogenous AARSs and tRNAs in the host, is used or evolved to direct the incorporation of a non-canonical amino acid (ncAA) into the specific site of target proteins (Mukai et al., 2017) (Figure 1). Because of diversified functional groups, ncAAs endow proteins with modified structures, functions, and interactions (O’Donoghue et al., 2013). This approach, named genetic code expansion, has paved the way for various applications in biological studies (Chin, 2014; Li and Liu, 2014; Neumann-Staubitz and Neumann, 2016). It has also been exploited to design new therapeutics and vaccines for pharmaceutical applications (Axup et al., 2012; Wang et al., 2014).

Recently, cell-free protein synthesis (CFPS) has stepped into the field of genetic code expansion (Hong et al., 2014). Even though with limitations such as the need for folding chaperones and the lack of natural membranes for membrane protein synthesis, CFPS also has a number of unique advantages over living cells for protein synthesis. First, the open system allows convenient manipulation of the protein synthesis environment and facilitates appropriate protein folding due to the considerable distance between the ribosomes (Rosenblum and Cooperman, 2014). Second, it permits the synthesis of proteins toxic to cells (Worst et al., 2015). Third, it has no limit for cellular uptake and solubility of target ncAAs (Bundy and Swartz, 2010). Fourth, CFPS does not need to grow, collect, and break cells to obtain target proteins, thus saving time for downstream experiments, which is especially important for unstable proteins.

## Cell-Free Protein Synthesis for Post-Translational Modifications

It becomes crucial to study post-translational modifications (PTMs) because of their significant role in various cellular functions and biological processes (Lothrop et al., 2013). Besides being mostly reversible, competitions among different PTMs for the same residues and limited knowledge of modifying enzymes make it difficult to synthesize homogeneously modified proteins for studying. To overcome these challenges, the genetic code expansion strategy has been applied in PTM studies (Liu et al., 2011; Chen et al., 2018). Here, we summarized the applications of CFPS in generating proteins with PTMs through the genetic code expansion strategy.

### Tyrosine Phosphorylation

The first *in vitro* work of site-specifically incorporating phosphotyrosine (pTyr) into proteins was performed to generate modified luciferase analogs (Arslan et al., 1997). In this work, pTyr was attached to the di-nucleotide pdCpA and then linked to the tRNA transcript lacking the last two nucleotides (CA) by T4 RNA ligase. The pTyr-charged tRNA with a CUA anticodon was used to suppress the UAG codon in the luciferase mRNA transcript to incorporate pTyr in the designed site of luciferase. The similar approach was also used to incorporate a pTyr analog, phosphonotyrosine into luciferase. However, the incorporation efficiency was relatively low. To increase the yield, a nitrophenylethyl-caged pTyr was synthesized to mask the negative charges of the phosphate as the elongation factor (EF-Tu) binds poorly with negatively charged amino acids (Rothman et al., 2005). The caging group could be easily removed by the UV light after incorporating into the protein. The same approach was also applied to generate phosphoserine (pSer)- and phosphothreonine (pThr)-modified proteins. By using vasodilator-stimulated phosphoprotein (VASP) that is involved in cell migration processes as a target protein, the phosphoprotein generated by CFPS was shown to be active as expected. Another analog of pTyr, phosphonoamino-phenylalanine, was later incorporated into protein by two steps. First, the azido-phenylalanine-modified protein was generated by CFPS with the amber-suppression system. Then, the azido-phenylalanine side chain was modified into phosphonoamino-phenylalanine through the Staudinger-phosphite reaction (Serwa et al., 2009).

### Serine Phosphorylation

It has been a challenge for decades to study serine phosphorylation both structurally and functionally. Although many attempts were made by using mimics such as aspartate or glutamate, they could not reflect the real situation due to charge and size differences. Efforts made to incorporate pSer *in vivo* by the genetic code expansion strategy have been successful (Park et al., 2011; Heinemann et al., 2012; Pirman et al., 2015; Rogerson et al., 2015). However, some issues such as endogenous phosphatases in host cells, which could remove phosphorylation, still exist.

In order to tackle these limitations, CFPS was applied to achieve pure and high yield of serine phosphorylation at multiple sites in proteins (Oza et al., 2015). One issue with the amber-suppression approach is the competition with the release factor-1 (RF1) for the stop codon. Thus, in this study, a genomically recoded RF1-deficient strain (*E. coli* C321.ΔA) in which all 321 TAG codons in the genome were substituted with TAA codons to improve cell growth and increase purity of ncAA incorporation (Lajoie et al., 2013) was used to generate the CFPS system. By using human mitogen-activated ERK activating kinase 1 (MEK1) as a validating example, 1 mg of phosphorylated MEK1 was obtained from 2 ml of CFPS reaction mixtures (Oza et al., 2015). This was a dramatic increase in protein yield compared to that of the *in vivo* pSer incorporation system in which only 1 μg of phosphorylated MEK1 was obtained from 1 L of cell culture (Park et al., 2011).

**Figure 2:**
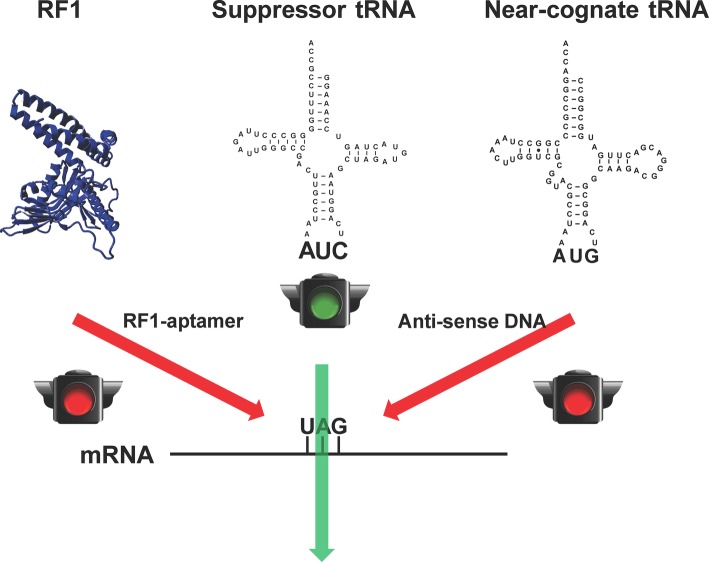
Increasing the fidelity of ncAA incorporation in CFPS. To remove competitions from the release factor-1 (RF1) and near-cognate tRNAs (tRNA^Tyr^ as a representative) for the amber stop codon UAG for ncAA incorporation, RF1-sepcific aptamer and near-cognate tRNA-specific antisense DNA oligos were used, respectively.

Although RF1-deficient systems could eliminate the early termination of target proteins in which the amber stop codon is used as the signal of ncAA incorporation, the suppression of the amber codon by endogenous near-cognate tRNAs could bring canonical amino acids into the desired site to cause impurity (Aerni et al., 2014). It is lethal to remove those near-cognate tRNAs from cells due to their essential role in normal protein synthesis. However, CFPS has no such limitations because all the translation components such as RFs and tRNAs can be modulated. Taking the advantage of CFPS, we have recently developed a method to increase the purity of pSer-containing proteins in the RF1-deficient background by reducing the amount of near-cognate tRNAs for the amber stop codon (Gan and Fan, 2017) (Figure 2). In this work, we first used an RF1-specific RNA aptamer to eliminate the RF1 activity in the CFPS system. Then, we applied antisense oligos to reduce the amount of near cognate tRNAs for the amber stop codon (tRNA^Tyr^, tRNA^Lys^, and one tRNA^Gln^ isoacceptor). Without affecting the protein yield, the purity of pSer incorporation at the amber codon increased significantly.

For phosphoprotein synthesis, one challenge of using living cells is the poor uptake of negatively charged phosphoamino acids. Even for phosphoserine that is an intermediate in serine biosynthesis, the concentration of the free amino acid is still relatively low in cells (Steinfeld et al., 2014). Another issue with living cells is endogenous phosphatases, making phosphoprotein dephosphorylated easily. CFPS is able to solve both problems ideally. First, CFPS is an open system and has no issue of amino acid uptake. Second, the PURE system contains only translation components without any phosphatases. Furthermore, enzymatic syntheses of pTyr- and pThr-charged tRNAs have been recently established (Fan et al., 2016; Zhang et al., 2017), making it convenient for biological laboratories to generate pTyr- and pThr-containing proteins without multi-step organic synthesis of pTyr- or pThr-charged tRNAs by chemical ligation.

### Lysine Acetylation

Lysine acetylation is one of the most well-studied PTMs because of its crucial role in DNA replication, DNA repair, and transcription (Sterner and Berger, 2000). To study lysine acetylation without concerning about endogenous deacetylases in cells, non-deacetylatable analogs of *N^ε^*-acetyllysine (AcK) such as *N^ε^*-thio-acetyllysine (ThioAcK) and *N^ε^*-seleno-acetyllysine (SeAcK) were proposed. ThioAcK, which has only a single atom replacement, has been shown to be the most comparable analog of AcK in structures and functions in our recent *in vivo* studies with the genetic code expansion strategy (Venkat et al., 2017).

Actually, ThioAcK has already been incorporated into proteins in a previous work by combining flexizyme and the PURE (protein synthesis using purified recombinant elements) system (Xiong et al., 2016). Flexizyme is a catalytic RNA, which is able to mediate the aminoacylation of the tRNA with a wide range of ncAAs, providing scientists with a facile method to obtain ncAA-charged tRNAs without labor-consuming AARS engineering (Morimoto et al., 2011). The PURE system contains affinity-purified protein components of the translational machinery including initiation factors, elongation factors, release factors, ribosome recycling factors, and 20 AARSs (Shimizu et al., 2001). In this work, flexizyme was first used to generate suppressor tRNAs charged with AcK, ThioAcK, or SeAcK, individually. Then, the aminoacylated suppressor tRNA was added into the PURE *ΔRF1* system to incorporate AcK or its analogs at K9 and K56 positions of histone H3. Results showed highly efficient incorporation at both positions. Furthermore, by using two suppressor tRNAs harboring different anticodons, the study also made the dual incorporation of AcK and ThioAcK in the same protein possible, paving the way for future studies that can lead to deeper understanding of the crosstalk between PTMs.

Besides flexizyme-mediated tRNA aminoacylation, enzyme-catalyzed tRNA charging by evolved pyrrolysyl-tRNA synthetase (PylRS) variants has also been used to generate site-specifically acetylated proteins such as histone H4 to study inter- and intra-nucleosome interactions by CFPS (Wakamori et al., 2015). As the charged tRNA^Pyl^ can be recycled for another round of tRNA aminoacylation, the protein yield was much higher than those generated by chemical or ribozyme-dependent aminoacylation. Advancements of CFPS for ncAA incorporation followed the development of RF1-deficient strains, which were designed to aid UAG codon reassignment. As the host strain for the CFPS system, BL21-based RF1-deficient cells were optimized for cell growth by engineering an essential gene *sucB* (Mukai et al., 2011). The optimized CFPS could generate full-length histone H4 with four lysine residue acetylated simultaneously with the yield of 0.2 mg protein per 1 ml reaction, while the yield of the same protein produced in living cells was 1 mg per 1 L culture.

### Lysine Methylation

Lysine methylation is another important PTM of lysine residues that modulates epigenetic statuses and various other biological processes including heterochromatin development and transcription (Lanouette et al., 2014). Different from lysine acetylation, lysine methylation has various forms including mono-, di-, and tri-methylation, which have different effects on protein functions (Martin and Zhang, 2005). Furthermore, due to the similarity between methylated lysine (MeK) and lysine itself, it is difficult to evolve AARSs specifically for MeK only. Thus, genetic incorporation of lysine methylation in living cells has not succeeded.

On the other hand, evolved AARSs for MeK incorporation are not necessary for CFPS, which has already been used to produce site-specifically methylated proteins. To introduce mono-methyllysine, di-methyllysine, and tri-methyllysine, frameshift and amber-suppressor tRNAs charged with MeKs were synthesized by chemical aminoacylation and supplied to an *E. coli* CFPS system (Tokuda et al., 2011). The MeKs were successfully incorporated into streptavidin and histone H3 in a quantitative, position-specific manner.

Similar to the AcK incorporation, RF1-deficient strains have also been used for MeK incorporation in CFPS (Yanagisawa et al., 2014). In order to study the impact of lysine methylation in histone H3, cell lysates of RF1-deficient strains were used to incorporate *N^ε^*-(*tert*-butyloxycarbonyl)-*N^ε^*-methyl-l-lysine (BocKme1), a protected mono-MeK analog, by an engineered PylRS variant first, which was then removed by trifluoroacetic acid. Up to four lysine residues could be modified at the same time by the CFPS system. Compared with the genetic incorporation in living cells, the CFPS system has an important advantage in which the amounts of proteins obtained per consumed BocKme1 were considerably larger (10–20 folds) than those of the cell-based system. This is also true for other cell-based ncAA incorporation in which a large proportion of ncAAs is wasted in cell growth media without efficient approaches to recycle. Thus, CFPS is a more cost-efficient choice when target ncAAs are expensive or need special synthesis.

## Conclusions

In this review, we summarized the applications of CFPS in generating proteins with PTMs by the genetic code expansion strategy. Generally, major differences between these approaches are the methods to get ncAA-charged suppressor tRNAs. The traditional chemical ligation method needs extensive organic synthesis, which could not be practical for biological laboratories. Flexizyme-mediated tRNA charging is relatively easy to handle and also could provide a wide range of ncAA candidates. However, suppressor tRNAs cannot be recycled, making the protein yield relatively low. Evolved AARSs for specific ncAAs could overcome this issue to increase the incorporation efficiency. But, AARS engineering is time- and labor-consuming and may not be successful. So, researchers should choose appropriate approaches, which fit for their own laboratory setup and project needs. There are also many other successful works of using CFPS to generate proteins with PTMs such as glycosylation (Tarui et al., 2001; Jaroentomeechai et al., 2018) and myristoylation (Suzuki et al., 2010; Yamauchi et al., 2010) through enzymatic modifications, which were not discussed in this review.

## Perspectives

CFPS has led to many break-through in various fields. In structural biology, CFPS could generate high yields of cytotoxic proteins for crystallization (Avenaud et al., 2004). CFPS can also increase the incorporation efficiency of selenomethionine into protein to help crystallographers with protein phasing (Kigawa et al., 2002). In the diagnostics and therapeutics, CFPS has been used for generating viral particles and detecting mutations in marker genes by coupling transcription and translation of specific RT-PCR or PCR amplified products (Cello et al., 2002; Gite et al., 2003). In the field of synthetic biology, CFPS has grown to emerge as an assuring approach for incorporating ncAAs at specific sites in proteins, setting the stage for studying protein interactions and PTMs. The course of development for CFPS in genetic code expansion would be prominently concentrated on building better genetic incorporation systems and more efficient cell extracts. This will not only be in terms of reducing the cost of the systems but also making it readily available to the ever-changing scientific needs.

A promising application of CFPS in PTM studies would be to generate a single protein with multiple PTMs simultaneously. Although most of studies generated proteins with only one type of PTMs, it is known that proteins usually have multiple PTMs, which co-regulate protein functions. Thus, it is necessary to generate multi-modified proteins. Recently, we have shown that the most-widely used genetic incorporation systems, which are derived from the *Methanocaldococcus jannaschii* tyrosyl-tRNA synthetase for incorporation of tyrosine or phenylalanine analogs, the *Methanosarcina* species PylRS for incorporation of lysine derivatives, or the *Methanococcus maripaludis* phosphoseryl-tRNA synthetase for pSer/pThr incorporation, are orthogonal to each other, making it possible to produce dual- or tri-modified proteins (Venkat et al., 2018). However, multiple ncAA incorporation in living cells needs to introduce a number of plasmids, which usually causes strong growth effects to lower protein yields. CFPS has no such concerns for cell growth; thus, it could be the first choice to generate proteins with multiple PTMs simultaneously.

## Author Contributions

All authors listed have made a substantial, direct and intellectual contribution to the work, and approved it for publication.

### Conflict of Interest Statement

The authors declare that the research was conducted in the absence of any commercial or financial relationships that could be construed as a potential conflict of interest.
